# Gun ownership for political protection or armed political expression: a nationally representative analysis of differences in 2025 vs. 2023

**DOI:** 10.1186/s40621-026-00655-8

**Published:** 2026-01-19

**Authors:** Julie A. Ward, Rebecca A. Valek, Vanya C. Jones, Lilliana Mason, Cassandra K. Crifasi

**Affiliations:** 1 Department of Medicine, Health, and Society, Vanderbilt College of Arts and Science, 2301 Vanderbilt Place, Nashville, TN 37235 USA; 2 Oregon Health & Science University, Portland State University School of Public Health, 1810 SW 5th Ave, Portland, OR 97201 USA; 3 Department of Health, Behavior and Society, Johns Hopkins Bloomberg School of Public Health, 624 N. Broadway, Baltimore, MD 21205 USA; 4 Center for Gun Violence Solutions, Johns Hopkins Bloomberg School of Public Health, 624 N. Broadway, Baltimore, MD 21205 USA; 5 SNF Agora Institute of Political Science, Johns Hopkins University, 3100 Wyman Park Dr, Baltimore, MD 21218 USA; 6 Department of Health Policy and Management, Johns Hopkins Bloomberg School of Public Health, 624 N. Broadway, Baltimore, MD 21205 USA

**Keywords:** Gun ownership, Political violence, Partisanship, Gun violence prevention, Public safety

## Abstract

**Background:**

Reasons for gun ownership have shifted from primarily for hunting, to protection from other people, and increasingly for concerns about political violence. In 2023, these reasons differed by party affiliation. In the aftermath of the 2024 Presidential election, the objective of this study was to compare gun owners’ reasons for gun ownership in January 2025 vs. 2023, overall and by political party affiliation.

**Methods:**

We analyzed two waves of gun owning respondents to the National Survey of Gun Policy (*n* = 2,003). In both waves, fielded 1/4/23 − 2/6/23 and 1/6/25 − 1/24/25, respondents identified personally important reasons for gun ownership from 10 potential reasons (e.g., at-home protection, out-of-home protection, protection from police, ideological conflict, hunting or recreation). We calculated weighted proportions to generate nationally representative estimates and compared reasons for gun ownership in 2025 to 2023 overall and by political affiliation (i.e., Republican, Democrat, or Independent).

**Results:**

In 2025 (vs. 2023), more gun owners valued gun ownership “for protection at demonstrations, rallies, or protests” (42% vs. 35%) and for hunting (81% vs. 74%), but fewer valued ownership “to advance an important political objective” (22% vs. 35%). Increases were largely driven by Republican gun owners, who also rated higher at-home protection (97% vs. 93%) and protection against police violence (34% vs. 25%). Fewer Republican, Democrat, and Independent gun owners valued ownership “to advance an important political objective.”

**Conclusions:**

As political violence escalated nationally, larger portions of gun owners rejected such violence, while also seeking to protect themselves from it. Safety and policy implications are discussed.

## Introduction

In 2023, an estimated 32% of US adults were gun owners, including 45% of Republicans and 20% of Democrats [1]. Reasons for gun ownership in the US have shifted over time, with more gun owners now citing protection against other people than hunting-related motivations [2]. More recently, fear of the government and perceived political threats have also increasingly been identified among gun owners’ reasons for ownership [3, 4]. In 2023, 59% of all gun owners – including 85% of new gun owners since January 2020 – identified at least one form of protection from ideological conflict among their personally held reasons for gun ownership [4]. Prior research suggests that differences in reasons for gun ownership are linked to and potentially directly influenced by political party affiliation [4, 5]. In 2023, 51% of Democrats cited ideological concerns among their reasons for gun ownership; significantly more Republican and Independent gun owners assigned importance to these reasons (61% and 62%, respectively) [4].

The implications of this observed growth in politically linked reasons for gun ownership are significant within the broader US social and political landscape. In 2024, 35% of Americans believed that civil war was imminent, with 12% strongly or very strongly agreeing that violent actions may be needed to protect “our American way of life” [6]. In 2023, 14% of gun owners reported supporting the principles of the Christian Nationalist movement [7], a movement characterized by beliefs in maintaining “cultural and blood purity, often through war, conquest, and separatism” [8]. Such affiliation has also been linked to opposition to stricter federal gun laws [9] and strong political endorsement of then-candidate Donald Trump [10].

On January 20, 2025, Mr. Trump was sworn in as the 47th President of the United States, following a tumultuous and violent election campaign, marked by the escalation of “aggressive” and “divisive” campaign language [11], verbal and physical confrontations with anti-Trump demonstrators at political rallies [12], and two assassination attempts [13]. Prompted by these events, assessed trends, and the transition in power from Democrat to Republican leadership, the objective of this study was to examine reasons for gun ownership among US gun owners in January 2025 as compared to 2023, both overall and by political party affiliation.

## Methods

### Sample and data collection

The National Survey of Gun Policy, fielded every two years using NORC’s Amerispeak Panel, assesses public support for gun policy and related issues [4, 7]. The Amerispeak Panel is a nationwide probability-based sample of US adults drawn from the US Postal Service Delivery Sequence File with enhancements for rural area coverage. The resulting sampling frame includes 97% of US households [14]. Waves 6 and 7 of the survey were fielded January 4, 2023–February 6, 2023 and January 6, 2025–January 24, 2025, respectively. To enable comparisons of under-represented groups, we oversampled Hispanic, African American, Asian, and gun owning adults. Gun owners were identified by affirmative responses to two questions: “Do you happen to have in your home or garage any guns or revolvers?” and “Do any of these guns personally belong to you?” Respondents could complete the survey online or by telephone in English or Spanish.

### Measures

Respondents who self-identified as gun owners were presented with a module assessing their personally held reasons for gun ownership. This module opened by stating, “People have guns for many reasons. These days, how important is each of the following reasons for you?” Subsequently, 10 potential reasons were presented in random order. These reasons related to in-home protection (i.e., “for protection from dangerous people or situations when I am at home”), out-of-home protection (i.e., “for protection from dangerous people or situations when I am away from home,” or “for protection at public events, such as sporting events or concerts”), protection from police (i.e., “for protection against police violence”), ideological conflict (i.e., “for protection during political activities, such as while voting or listening to speeches,” “for protection at demonstrations, rallies, or protests,” “for protection against people who do not share my beliefs,” or “for situations where I think that force or violence is justified to advance an important political objective”), and hunting or recreational reasons (i.e., “for hunting” or “for other recreational activities, such as target shooting”). Respondents were asked to rate each reason as “not important,” “somewhat important,” “very important,” or “extremely important.” Ratings of importance were dichotomized to represent “not important” vs. “important” (comprised of “somewhat,” “very,” or “extremely”).

Respondent demographics included political party affiliation, for which respondents could self-describe as Democrat or Republican on a 7-point scale. Political party affiliation was subsequently collapsed into three classifications: Republican (i.e., “strong” “not so strong” or “lean” Republican), Democrat (i.e., “strong” “not so strong” or “lean” Democrat), and Independent (i.e., “Don’t Lean/Independent/None”). Other assessed demographics included sex, age, race and ethnicity, education, household income, employment status, and US region of residence.

### Analysis

The study sample was restricted to gun owning respondents of the 2023 and 2025 surveys (*n* = 1,002 and *n* = 1,001, respectively). Respondents from the 2025 survey were designated 1 (0 otherwise) to enable comparisons between survey waves. Next, we applied 2025 survey weights to the full sample to calculate nationally representative estimates of the US gun owning population. For each reason for gun ownership, we calculated weighted proportions of agreement with 95% confidence intervals in 2023 and 2025 overall and stratified by party affiliation. Logistic regression models were estimated to designate statistically significant differences in proportionate importance in 2025 (vs. 2023) based on an alpha of 0.05. Analyses were conducted using the *svy* command in Stata, version 16.1. The study was reviewed and approved by the Johns Hopkins Bloomberg School of Public Health IRB.

## Results

The total sample of US gun owners (*n* = 2,003) was distributed evenly across survey years (Table 1). Of the 10 assessed reasons for gun ownership, 3 reasons were assigned importance by a statistically significantly larger or smaller proportion of 2025 (vs. 2023) gun owners. Specifically, 42% of gun owners in 2025 assigned importance to owning guns “for protection at demonstrations, rallies, or protests,” compared with 35% in 2023 (*p* = 0.009). Conversely, fewer gun owners in 2025 assigned importance to gun ownership “for situations where I think that force or violence is justified to advance an important political objective” (2023: 34% vs. 2025: 22%, *p* < 0.001). Additionally, we observed larger agreement with owning guns for hunting (2023: 74% vs. 2025: 81%, *p* = 0.008) (Table 2).

**Table 1:** Weighted and unweighted demographic characteristics of the study samples, 2023 and 2025

			2023 Unweighted *n* = 1,002 (%)	2025 Unweighted *n* = 1,001 (%)	2023 and 2025 Weighted %
Sex					
Male			707 (70.6)	678 (67.7)	63.9
Female			295 (29.4)	323 (32.3)	36.1
Age					
18–29			73 (7.3)	70 (7.0)	11.1
30–44			251 (25.1)	229 (22.9)	25.4
45–59			246 (24.6)	268 (26.8)	28.2
60+			432 (43.1)	434 (43.4)	35.3
Race and Ethnicity					
Non-Hispanic White			597 (59.6)	563 (56.2)	70.6
Non-Hispanic Black			177 (17.7)	220 (22.0)	11.9
Asian or Pacific Islander			43 (4.3)	50 (5.0)	2.9
Hispanic			153 (15.3)	142 (14.2)	11.8
Other or Multiple			32 (3.2)	26 (2.6)	2.9
Education					
High school diploma or less			201 (20.1)	218 (21.8)	28.8
Some college			422 (42.1)	492 (49.2)	35.9
Bachelor’s degree or higher			379 (37.8)	291 (29.1)	35.3
Household income					
<$30,000			148 (14.8)	163 (16.3)	14.9
$30,000 - <$60,000			269 (26.9)	251 (25.1)	25.7
$60,000 - <$100,000			285 (28.4)	289 (28.9)	29.1
$100,000 or more			300 (29.9)	298 (29.8)	30.2
Employment status					
Employed			610 (60.9)	606 (60.5)	64.6
Unemployed			127 (12.7)	110 (11.0)	12.7
Retired			265 (26.5)	285 (28.5)	22.7
Region					
Northeast			92 (9.2)	89 (8.9)	14.0
Midwest			259 (25.9)	246 (24.6)	21.3
South			442 (44.1)	448 (44.8)	47.7
West			209 (20.9)	218 (21.8)	17.0
Political Party Affiliation					
Republican			472 (47.1)	461 (46.1)	51.0
Independent			161 (16.1)	162 (16.2)	16.5
Democrat			369 (36.8)	374 (37.4)	32.3
Missing			0 (0)	4 (0.4)	0.3

Weighted N = 2,003

**Table 2:** Changes in US gun owners’ personally important reasons for gun ownership, 2023–2025 (weighted)

Reason for ownership^1^	Overall			Republican			Independent			Democrat		
	2023 *n* = 997.4 % (95% CI)	2025 *n* = 996.5 % (95% CI)	*p* value	2023 *n* = 500.6 % (95% CI)	2025 *n =* 518.5 % (95% CI)	*p* value	2023 *n* = 163.8 % (95% CI)	2025 *n* = 165.4 % (95% CI)	*p* value	2023 *n* = 333.0 % (95% CI)	2025 *n =* 312.6 % (95% CI)	*p* value
**For protection from dangerous people or situations when I am at home**	91.6 (89.1, 93.5)	93.0 (90.5, 94.8)	0.368	**92.7** (88.9, 95.3)	**97.1** (94.4, 98.5)	**0.021**	94.9 (88.1, 97.9)	91.4 (83.9, 95.6)	0.345	88.1 (83.5, 91.6)	87.9 (82.2, 92.0)	0.949
**For protection from dangerous people or situations when I am away from home**	82.0 (78.7, 84.8)	81.6 (78.2, 84.5)	0.873	87.6 (83.2, 91.0)	90.9 (87.0, 93.7)	0.208	82.9 (73.8, 89.2)	75.7 (65.1, 83.9)	0.250	73.0 (66.9, 78.4)	70.0 (63.6, 75.7)	0.476
**For protection at public events**,** such as sporting events or concerts**	43.5 (39.6, 47.4)	46.1 (42.0, 50.3)	0.359	50.7 (45.1, 56.2)	55.9 (49.7, 61.9)	0.215	44.3 (34.4, 54.6)	44.4 (34.8, 54.4)	0.987	32.2 (26.1, 39.0)	31.1 (25.3, 37.5)	0.808
**For protection against police violence**	30.3 (26.8, 34.1)	34.3 (30.5, 38.3)	0.149	**24.6** (20.1, 29.8)	**34.0** (28.4, 40.0)	**0.016**	40.9 (31.6, 50.9)	39.1 (29.9, 49.2)	0.802	33.6 (27.5, 40.3)	32.7 (26.7, 39.3)	0.845
**For protection during political activities**,** such as while voting or listening go speeches**	32.3 (28.7, 36.1)	36.9 (33.0, 41.0)	0.094	35.7 (30.6, 41.2)	43.5 (37.6, 49.5)	0.058	33.0 (24.4, 43.0)	38.4 (29.1, 48.6)	0.441	26.8 (21.0, 33.4)	25.7 (20.4, 32.0)	0.814
**For protection at demonstrations**,** rallies**,** or protests**	**34.5** (30.8, 38.4)	**41.9** (37.9, 46.1)	**0.009**	**40.3** (35.0, 46.0)	**50.5** (44.4, 56.6)	**0.017**	34.1 (25.1, 44.3)	42.6 (33.0, 52.8)	0.232	25.9 (20.3, 32.4)	28.0 (22.6, 34.1)	0.629
**For protection against people who do not share my beliefs**	29.4 (25.9, 33.1)	29.3 (25.6, 33.3)	0.979	31.3 (26.4, 36.6)	33.7 (28.1, 39.9)	0.536	29.2 (21.1, 38.9)	30.3 (21.6, 40.6)	0.874	26.6 (20.9, 33.1)	21.8 (16.7, 27.9)	0.255
**For situations where I think that force or violence is justified to advance an important political objective**	**34.2** (30.5, 38.0)	**22.0** (18.8, 25.5)	**< 0.001**	**33.2** (28.3, 38.6)	**23.9** (19.1, 29.5)	**0.015**	**39.8** (30.4, 50.0)	**20.2** (13.9, 28.3)	**0.002**	**32.8** (26.8, 39.5)	**19.9** (15.1, 25.7)	**0.003**
**For hunting**	**74.2** (70.6, 77.4)	**80.6** (77.1, 83.6)	**0.008**	**81.0** (76.3, 85.0)	**87.9** (83.6, 91.2)	**0.022**	71.4 (61.2, 79.8)	76.1 (66.2, 83.8)	0.478	65.3 (58.9, 71.2)	70.3 (63.6, 76.2)	0.269
**For other recreational activities**,** such as target shooting**	88.1 (85.2, 90.5)	90.6 (88.1, 92.6)	0.162	93.3 (89.5, 95.7)	95.2 (92.4, 97.0)	0.303	87.0 (76.4, 93.3)	91.3 (83.2, 95.7)	0.399	80.9 (75.3, 85.5)	82.5 (76.7, 87.1)	0.668

Statistically significant difference indicated in bold

^1^respondents could designate multiple reasons as personally important

Stratified by party identity, more widespread shifts in reasons for gun ownership were observed among Republican gun owners than gun owners who identified as Independent or Democrat. More Republican gun owners in 2025 assigned importance to protection at home (2023: 93% vs. 2025: 97%, *p* = 0.021), protection against police violence (2023: 25% vs. 2025: 34%, *p* = 0.016), protection at demonstrations, rallies, or protests (2023: 40% vs. 2025: 51%, *p* = 0.017), and hunting (2023: 81% vs. 2025: 88%, *p* = 0.022). Across Republican, Independent, and Democrat gun owners, we observed a decrease in assigned importance “to advance an important political objective.” In 2025, 24% of Republican gun owners agreed (vs. 2023: 33%, *p* = 0.015), 20% of Independent gun owners agreed (vs. 2023: 40%, *p* = 0.002), and 20% of Democrat gun owners agreed (vs. 2023: 33%, *p* = 0.003). No other reasons for ownership were statistically significantly more or less important in 2025 vs. 2023 for Democrats and Independents (Table 2).

## Discussion

Among US gun owners overall, we observed diffuse but generally stable reasons for gun ownership in January 2025 compared with January 2023. Two significant exceptions included more gun owners in 2025 endorsing protection at political events, and fewer assigning personal importance to gun ownership for advancing a political objective. The first shift appeared to largely be driven by Republican gun owners, for whom multiple reasons for gun ownership saw gains. The largest of these gains, protection at political events, was unique to the Republican respondent group, a group whose rallies were sites of notable political violence in the 2024 campaign season [12]. In contrast, our finding of correspondingly lower agreement with owning guns for advancing a political objective was observed across all political affiliations. Gun ownership for hunting-related reasons also increased overall and among Republican gun owners specifically.

Prior research suggests that gun-related recreation and threat perception may work synergistically to influence gun ownership in potentially partisan ways [5]. Other potentially partisan determinants of gun ownership may include political messaging [15], industry marketing [16], concerns about religious or political security [5], or perceived threats to social standing [17]. Though speculative, the co-occurring, partisan increase in hunting-related and protection-related motivations for gun ownership may further be shaped and reinforced by algorithm-driven social media “gunfluencers,” and other similar marketing tactics, which have been observed to embed protection-related beliefs within a normalized identity- and consumption-oriented culture [18]. News media exposure and trust in law enforcement may further shape patterns of perceived threat and reasons for gun ownership. Prior research identified a positive association between news media consumption and protective gun ownership, mediated by the belief in a dangerous world; lower trust in law enforcement was also associated with higher support for protective gun ownership [19]. This study found that protection from police violence was a fourth reason for gun ownership that received higher endorsement from Republican gun owners in 2025 than 2023, largely mirroring the 2025 responses of Independent- and Democrat-affiliated gun owners.

Our findings differ somewhat from Wintemute et al.’s observations of generally low and stable endorsement of violence “to advance an important political objective” among a nationally representative sample in May-June of 2024 vs. 2023 and 2022 [6]. However, Wintemute et al.’s national study was not specific to gun owners, and it was fielded relatively early in a contentious election year. In another nationally representative survey, fielded concurrently with the first assassination attempt in July 2024, Holliday et al. observed significant post-attempt reductions in Republican support for partisan violence [20]. Views on gun ownership may be similarly sensitive to such extraordinary acts of violence.

Guns and gun policy have become an issue of durable political prominence in the US, such that public attention, political activism, and oppositional backlash are no longer confined to a temporary news cycle. Rather, they have become structurally “baked into” US politics [15]. Goss and Lacombe theorize that two influential factors in establishing such prominence may be the recurrence of gun-involved threats—making the issue feel personal—and partisan polarization, which can increase activism and intensify an issue’s perceived stakes [15]. Further, their research found that since 2010, pro-gun views have increasingly “lost their dominance” in US gun politics [15]. Accordingly, our research finds that as political violence escalates nationally, a larger portion of gun owners appear to both be rejecting such acts of violence and seeking to protect themselves from it. Future research should explore whether such views on gun ownership are associated with support for policies to protect people from risk for gun violence in politically sensitive places.

### Limitations

The results of this study should be interpreted in the context of some limitations. First, we compared two cross-sectional waves of a nationally representative survey. Patterns described should not be interpreted as changes among individual gun owners over time. Second, we were unable to assess the extent to which results may be time sensitive or otherwise linked to concurrent historical events. Future research should examine the persistence of political motivations for gun ownership, including beliefs about perpetrating versus defending against political violence. We also note that within-group variation may exist; we were unable to assess political subgroup identities in this analysis. As with any survey, risk for sampling bias should be considered. NORC’s probability-based sampling and survey weights serve to mitigate this risk. Given the sensitive nature of firearm ownership and related motivations, potential for social desirability bias remains.

### Implications

Amid concerns about escalating political violence, we identified that fewer gun owners agreed with owning guns for advancing a personally important political objective. That this finding was observed across all political affiliations is assuring for public safety and political expression. However, among Republican gun owners, we simultaneously observed increases in the perceived importance of ownership for protection across multiple contexts, including at demonstrations, rallies, or protests. Increased gun carrying, even when motivated by defensive interests, paradoxically increases risk for gun-related harms [21]. Policies to limit carrying in sensitive spaces, through the designation of gun-free zones, and implementation of permit-based concealed carry more broadly may better address underlying protection-seeking interests. Such assurances and safeguards may be particularly urgent in forthcoming election seasons.

## Data Availability

The datasets analyzed in the current study are available from the corresponding author upon reasonable request.
